# Th1/Th17-linked transcription factors TBX21 and RORC were associated with clinical response to rituximab in treatment-resistant schizophrenia

**DOI:** 10.1016/j.bbih.2026.101270

**Published:** 2026-05-27

**Authors:** Simon Bylund, Daniel Eklund, Mats B. Humble, Susanne Bejerot, Sverre Wikström

**Affiliations:** a School of Medical Sciences, Faculty of Medicine and Health, Örebro University, Sweden Örebro University Hospital, Region Örebro County, Sweden; b School of Medical Sciences, Faculty of Medicine and Health, Örebro University, Sweden; c School of Medical Sciences, Faculty of Medicine and Health, Örebro University, Örebro, Sweden; d University Health Care Research Center, Faculty of Medicine and Health, Örebro University, Örebro, Sweden; e Centre for Clinical Research, County Council of Värmland, Karlstad, Sweden

## Abstract

**Background:**

One third of patients with schizophrenia do not achieve remission with current antipsychotic treatments. Converging data support an immune-mediated component in a subset of cases, overlapping with autoimmune CNS disorders. The B-cell–depleting monoclonal antibody rituximab is used in diseases such as multiple sclerosis and has been explored as an immunomodulatory strategy. We investigated plasma biomarkers, focusing on T-helper–related transcription factors, in relation to outcome after rituximab in schizophrenia.

**Methods:**

Nineteen severely ill, treatment-resistant patients (n = 9 with schizophrenia and n = 10 with obsessive–compulsive disorder [OCD]) included in an open-label pilot trial, received a single 1000 mg intravenous infusion of rituximab. Symptoms were assessed at baseline and week 12 using PANSS or Y-BOCS, global functioning with PSP, and improvement with CGI-I. Response was defined as “much” or “very much improved” on CGI-I at week 12. For the present analyses, we compared schizophrenia responders with a combined group of schizophrenia and OCD non-responders. Plasma and whole blood were collected at baseline and week 12. Multiplex cytokines and RT-qPCR for the expression of 13 genes coding for cytokines and T-cell–related transcription factors were analyzed, including Th1 and Th17 master regulators TBX21 (T-bet) and RORC (RORγt), respectively. We also constructed a composite rank score combining TBX21 and RORC expression (sum of their within-sample ranks).

**Results:**

Six out of eight schizophrenia patients were classified as responders and showed large symptomatic improvement (median PANSS change −70.1) and marked functional gain (median PSP +22.5). Within the analytic sample, schizophrenia responders (n = 6) had higher baseline TBX21 and RORC expression than non-responders (n = 8). Baseline TBX21 correlated with PSP improvement at week 12 (r_s_ = 0.675, p < 0.01). The TBX21+RORC composite rank score showed the strongest association with outcome (higher in responders, p < 0.001; correlated with PSP gain, r_s_ = 0.793, p < 0.001). Classical inflammatory markers, including IL-6, TNF-α and high-sensitivity C-reactive protein, did not differentiate responders.

**Discussion:**

Elevated baseline expression of the Th1- and Th17-associated transcription factors TBX21 and RORC – particularly their combined composite rank score – was associated with symptomatic and functional response to rituximab in treatment-resistant schizophrenia. The data suggest that a TBX21/RORC-defined, Th1/Th17-skewed T-cell signature may characterize a rituximab-responsive immunopsychiatric subtype of schizophrenia, in which B-cell depletion may secondarily modulate pathogenic T-cell response. These findings should be viewed as preliminary and hypothesis-generating for future controlled studies.

## 1 Introduction

Approximately 30% of patients with schizophrenia do not achieve remission with current antipsychotic treatments (Siskind et al., 2022; Leung et al., 2023). Increasing evidence implicates immune dysregulation and neuroinflammation in the pathophysiology of schizophrenia and related disorders, suggesting that immunomodulatory strategies may offer therapeutic benefit (Khandaker et al., 2015; Attwells et al., 2017).

Elevated peripheral cytokines such as IL-6, IL-8, IL-1β, TNF-α, IFN-γ, and TGF-β have been repeatedly observed in schizophrenia and correlate with symptom severity (Goldsmith et al., 2016; Dawidowski et al., 2021; Halstead et al., 2023). Previous genome-wide association studies further linked schizophrenia risk loci to immune-related genes, particularly in B-lymphocyte lineages (Schizophrenia Working Group of the Psychiatric Genomics Consortium, 2014), implicating adaptive immune mechanisms.

Several anti-inflammatory agents have been evaluated as adjunctive treatments in schizophrenia, including aspirin, celecoxib, minocycline and omega-3 fatty acids. Although some studies report modest improvements, overall results remain inconsistent (Çakici et al., 2019).

Only a few trials have investigated monoclonal antibodies to modulate immune pathways in schizophrenia spectrum disorders. The IL-6 receptor antagonist tocilizumab showed no significant effect on symptoms or cognition in a randomized placebo-controlled trial (Girgis et al., 2018). Adalimumab, a TNF-antagonist, showed effect on negative symptoms (Motamed et al., 2022). More recently, the IL-1β antibody canakinumab reduced peripheral inflammation and modestly improved positive symptoms in patients with elevated inflammatory markers (Weickert et al., 2024).

The B-cell depleting monoclonal antibody rituximab, targeting the CD20 surface antigen, is well tolerated (Edwards et al., 2004) and widely used in autoimmune and neuroinflammatory diseases. In autoimmune encephalitis, rituximab is established as an effective second-line therapy, with meta-analyses reporting favorable outcomes and acceptable safety, though psychiatric and cognitive symptoms may persist (Nepal et al., 2020; Halliday et al., 2022; Dos Passos et al., 2016). Its therapeutic effect in multiple sclerosis is thought to derive from modulation of B-cell cytokine secretion and antigen presentation rather than direct antibody depletion, thereby attenuating downstream T-cell activation (Hauser et al., 2008). We have hypothesised that a similar mechanism is relevant in schizophrenia treatment (Bejerot et al., 2023a, 2023b; Humble et al., 2025; Humble et al., 2025b). Beyond B-cell dysregulation, converging evidence also implicates maladaptive T-cell responses in schizophrenia, in particular changes in CD4^+^ T cell populations. Impaired regulatory T-cell (Treg) function has been proposed to link low-grade peripheral inflammation with astrocytic and microglial activation in the CNS (Corsi-Zuelli and Deakin, 2021; Komori et al., 2015; Huppert et al., 2010), while complementary data indicate aberrant Th17 activity and related cytokine signatures in first-episode psychosis, schizophrenia, depression and bipolar disorder with Th1/Th17/Treg patterns correlating with clinical symptom dimensions (Noto MN et al., 2019; Debnath, M., & Berk, M. 2014; Beurel and Lowell, 2018; Borovcanin et al., 2020).

We have previously reported a case of full recovery following rituximab in a patient with pediatric autoimmune neuropsychiatric disorder associated with streptococcal infection (PANDAS; Bejerot et al., 2019) and open-label pilot studies in treatment-resistant schizophrenia and obsessive compulsive disorder (OCD; Bejerot et al., 2023a). In the pilot on schizophrenia, a single 1000 mg rituximab infusion added to treatment as usual was followed by a mean Positive and Negative Syndrome Scale (PANSS) reduction of about 37% at week 12 (absolute change −36.8 points), and 7 of 9 schizophrenia patients reached ≥40% symptom reduction, with improvements largely maintained up to 40 weeks; disability also improved, with Personal and Social Performance Scale (PSP) scores increasing from 32 to 42.5 at week 12. In contrast, the OCD cohort showed modest mean reductions in Yale-Brown Obsessive Compulsive Scale (Y-BOCS) scores (∼16 – 19%) and only 2 of 10 patients fulfilled the ≥35% response criterion, with limited functional gains (Bejerot et al., 2023a).

As seven of the nine patients with schizophrenia in the pilot study showed significant improvement after rituximab, we wanted to explore putative immune markers related to B-cell/T-cell interactions that could be correlated to this response.

### 1.1 Aim

Here we present an exploratory analysis of biomarkers in relation to response in our previously reported open-label pilot study.

The aim was to evaluate associations between baseline immune biomarkers and clinical response following rituximab in treatment-resistant schizophrenia, and also dynamic changes of these biomarkers by week 12 after treatment.

## 2 Methods

### 2.1 Study design and participants

Nineteen treatment-resistant patients (18-40 years) were recruited from psychiatric clinics, and diagnoses were confirmed using the DSM-5 criteria for schizophrenia and OCD, respectively. Full inclusion/exclusion criteria, diagnostic procedures, recruitment and rituximab administration logistics (premedication, infusion setting/monitoring) were previously described (Bejerot et al., 2023a). Nine participants had schizophrenia with severe symptoms as assessed by the positive and negative syndrome scale (PANSS, Kay et al., 1987). Ten had OCD and with severe symptoms as assessed by Yale-Brown Obsessive-Compulsive Scale (Y-BOCS, Goodman et al., 1989).

Rituximab was administered as add-on treatment to stable ongoing psychiatric medication. The treatment regimen consisted of a single 1000 mg intravenous rituximab infusion over 3–4 h, preceded by oral paracetamol, desloratadine, and betamethasone. Participants were followed for 12 months with repeated safety and efficacy assessments.

#### 2.1.1 Outcomes and responder definitions

The primary endpoint was improvement on the 7-point Clinical Global Impressions-Improvement Scale (CGI-I, Guy, 1976) at week 12. All patients were assessed by the same clinician. Response was defined as “much improved” or very “much improved” by week 12 as an appropriate time point for evaluating clinical response. Participants not responding during the entire trial were considered non-responders in the present analyses. Schizophrenia participants with response later than 12 weeks and the two responding OCD participants were excluded from the present analyses in order to make a clear distinction between response in schizophrenia participants and the non-response comparison group.

As only two schizophrenia participants met criteria for non-response at week 12, the primary exploratory comparison was conducted between schizophrenia responders and a combined non-responder group consisting of schizophrenia and OCD non-responders. This approach was chosen pragmatically to enable comparison with a non-responder group within the constraints of the present pilot sample. Complementary sensitivity analyses restricted to the schizophrenia subgroup were also performed, although these were limited by the small number of schizophrenia non-responders.

Secondary outcomes included change in global functioning, measured by the Personal and Social Performance Scale (PSP) from baseline to week 12. PSP has been used to assess disease burden in both schizophrenia and OCD trials (Morosini et al., 2000; Mavrogiorgou et al., 2016).

### 2.2 Sample collection

Peripheral blood samples were collected from fasting participants at two time points: baseline (week 0) and week 12. Blood was drawn in the morning between 8:00 and 10:00 a.m. to minimize diurnal variation in immune marker levels. Plasma samples were collected into EDTA-coated tubes, while samples for downstream gene expression analysis were drawn in PAXgene® tubes (PreAnalytix, Hombrechtikon, Switzerland). Each participant provided approximately 10 mL of whole blood at each collection point.

The collected samples were analyzed in the same batches, with both baseline and week 12 samples from each participant processed under the same experimental conditions to control for any potential technical variability.

### 2.3 Clinical routine parameters

Clinical routine parameters including circulating cell numbers and circulating immunoglobulins, were analyzed at the department of clinical chemistry at Örebro university hospital.

### 2.4 RNA preparation

Total RNA was isolated from whole blood using PAXgene tubes and the PAXgene® Blood RNA Kit according to the manufacturer's instructions. After isolation, the RNA sample was eluted in 80 μL of the supplied elution buffer. RNA was quantified by measuring the RNA (A260) concentration and sample purity (260/280 ratio) with a Thermo Scientific NanoDrop 2000 (Fisher Scientific, Göteborg, Sweden). The samples were stored at −80 °C before cDNA synthesis.

### 2.5 cDNA synthesis and RT-PCR

The High Capacity cDNA Reverse Transcription Kit (Applied Biosystems, Foster City, CA, USA) was used to synthesise the cDNA, according to the manufacturer's instructions. The template was 100 ng RNA in each reaction. Quantitative PCR was performed using a Quantstudio 7 Flex Real-Time PCR system (Applied Biosystems) with commercially available fluorescent probes from the same company (Taq-Man Gene Expression Assays, Supplemental Table 2). To enable analysis of general inflammatory cytokines, a total of nine cytokine genes were selected.

To analyse changes in T-cells between responders and non-responders, four transcription factors known to regulate Th1/Th2/Th17/Treg respectively were selected (Kempe et al., 2018).

The PCR reagents and 384-well plates were obtained from Applied Biosystems. Crossing threshold (Ct) values were calculated by Quantstudio 7 Flex Real-Time PCR system software using the second-derivative maximum method. All samples were analyzed in duplicates and the mean Ct values were used in further data analysis. Cycle threshold (Ct) cut-off value was set to 35 and all reactions had an efficiency between 90 and 110%. Samples where duplicates displayed a coefficient of variation (CV) > 0.2 was reanalyzed. The Ct method was used for the quantification of relative gene expression. TBP and HPRT1 were both evaluated as reference genes, where TBP was selected for normalization, determined by using the NormFinder R package (MOMA, Aarhus University Hospital, Denmark). The untreated (week 0) samples were used as calibrators for calculating the relative expression values following treatment using the delta-delta method (2-ΔΔCt). The Ct-value of IL-17A was above 35 in all included samples and was thus removed in subsequent analyses.

### 2.6 Measurement of cytokines and chemokines in plasma

Quantification of soluble cytokines in plasma was performed by using Multiplex bead technology (MILLIPLEX® MAP Kit, Human Cytokine/Chemokine Magnetic Bead Panels) according to the manufacturer's description (Merck Millipore, Burlington, MA, USA). Samples were analyzed on a Bio-Plex® 200 instrument (Bio-RAD, Hercules, CA, US) and Bio-Plex manager software version 6.2 were used to obtain all data. Results were derived through comparison to a standard curve (5-parameter logistic curve fit) of known concentrations of each analyte. Samples were analyzed in duplicates and the levels of different proteins were expressed as pg/mL. The analyzed cytokines included detection limit (pg/ml); mean assay coefficient of variation (%)): IFNℽ (1.3 pg/ml; 4.65%), IL-1Ra (1.6 pg/ml; 3.54%), IL-6 (0.6 pg/ml, 4.13%) CXCL8 (0.6 pg/ml; 4.38%), IL-10 (2.6 pg/ml; 4.37%), IL-17A (1.3 pg/ml; 5.15%), IL-18 lowest detection limit: (0.6 pg/ml; 4.01%), LT-α (1.6 pg/ml; 5.04%) and TNF (0.5 pg/ml; 8.8%). Values under limit of detection (LOD) were assigned half the threshold value in each sample.

#### 2.6.1 Statistical analysis

Differences in baseline biomarker levels between responders and non-responders, as well as between the schizophrenia and OCD groups, were analyzed using Mann-Whitney *U* test for non-parametric data. Tests for linear associations of biomarkers and functional improvement as rated with the Personal and Social Performance Scale. Continuous covariates were analyzed with Spearman's rank correlations and dichotomous variables with Mann–Whitney U-tests. The same approach was used to examine associations between these covariates and outcome variables (response status and PSP change). For an evaluation of potential confounding, and our sample being too small for multiple regression modelling, we conducted additional post-hoc univariate analyses in which TBX21, RORC and their composite rank-sum variable were related to candidate covariates (age, sex, BMI, smoking status and current antipsychotic treatment). To account for multiple comparisons, a False Discovery Rate (FDR) correction was applied post hoc using the Benjamini-Hochberg method.

Statistical significance was set at p < 0.05 for all tests and all p-values <0.1 were regarded as indicators of potential associations.

All analyses were performed using SPSS version 25.

## 3 Results

### 3.1 Participant characteristics

**Fig. 1:**
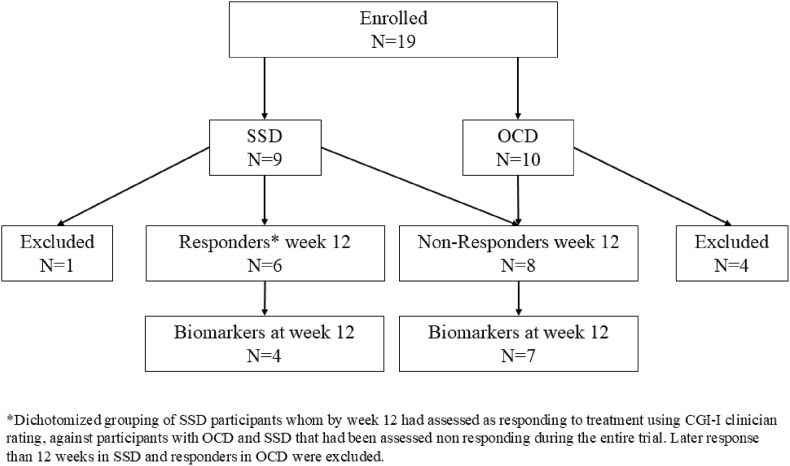
Flowchart of inclusion.

From the nineteen participants in the open-label pilot study, one participant in the schizophrenia group was excluded due to response after 20 weeks, and four participants in the OCD group were excluded as they met response criteria at week 12 (n = 2) or later (n = 2), hence not considered non-responders according to our definition (Fig. 1.).

The mean duration of illness was 13 years in the OCD group and 14 years in the schizophrenia group. At baseline, the schizophrenia group had a median PANSS total score of 105 (range 52–137), indicating severe symptomatology, while the OCD group had a median Y-BOCS score of 28.5 (range 15–37).

At 12 weeks, only 15 patients left blood samples, whereof 7 in the schizophrenia group (4 female) and 8 in the OCD group (6 female).

### 3.2 Baseline differences

In the schizophrenia group, six were classified as responders and showed a marked reduction in symptom severity, with a median decrease in PANSS total score of 70.1 points (range −84.6 to −49.0) at week 12. In contrast, non-responders (n = 2) exhibited a median decrease of 23.5 points (range −66 to −19).

Overall functional outcomes, assessed using the Personal and Social Performance (PSP) scale, also displayed a significant improvement in the schizophrenia group compared to the OCD (p = 0.004). The median PSP score increased by 22.5 points (range 20 – 45) in schizophrenia versus 2 points (range −5 to 6) in OCD (see Table 1).

When comparing biomarkers between the here included schizophrenia and OCD participants, we observed significant group differences with higher eosinophil counts and lower platelet (thrombocyte) counts among schizophrenia participants, together with higher TBX21 gene expression in schizophrenia (Table 2).

**Table 1:** Descriptives of the study group.

Diagnosis	Sex	Age at inclusion	Age at onset^b^	BMI	Smoking	Alcohol use	PANSS/Y-BOCS^a^	PANSS/Y-BOCS % (week 12)	PSP	PSP % (week 12)	Responders
Schizophrenia^c^	Female	39	8	54	No	No	52	−18	40	45	Response
Schizophrenia	Male	25	6	25.5	No	Yes	77	−35	31	20	Response
Schizophrenia	Female	22	6	20.6	No	No	129	−65	25	23	Response
Schizophrenia^c^	Male	26	8	20.9	No	No	134	−51	16	25	Response
Schizophrenia	Male	23	14	27	Yes	Yes	85	−36	41	22	Response
Schizophrenia	Female	25	20	17.4	No	No	69	−33	45	20	Response
Schizophrenia	Male	34	27	25.6	No	Yes	125	−66	28	22	Non-Response
Schizophrenia	Female	33	26	27.9	Yes	Yes	137	−19	21	4	Non-Response
OCD	Female	31	10	25.3	No	No	37	0	35	5	Non-Response
OCD	Female	23	11	26.3	Yes	Yes	31	−6	45	6	Non-Response
OCD	Female	24	14	40.7	No	No	24	−4	40	2	Non-Response
OCD	Female	19	14	23.1	No	No	23	9	45	−3	Non-Response
OCD^c^	Female	24	18	25.5	No	No	33	−18	35	−5	Non-Response
OCD	Female	32	8	23.1	No	No	33	0	30	−5	Non-Response

OCD	Male	36	16	25.6	No	Yes	15	−20	66	5	Excluded
Schizophrenia	Female	19	16	37.5	Yes	Yes	83	−8	41	4	Excluded
OCD	Male	26	12	23.4	No	No	28	−7	40	31	Excluded
OCD	Male	27	22	25.1	No	Yes	29	−31	41	24	Excluded
OCD^c^	Female	20	9	23.4	Yes	No	22	−82	48	3	Excluded

Descriptive statistics and possible confounders of the used study sample.

a Baseline value of PANSS of participants with the diagnosis of schizophrenia, Y-BOCS baseline value of participants with OCD.

b Onset of psychiatric symptoms

c Missing biomarkers collected at week 12.

**Table 2:** Baseline differences between the schizophrenia and OCD group.

	Schizophrenia (N = 8)	OCD (N = 6)	p-value
**Clinical routine measurements**			
Leukocytes	6.45 (4.40–11.80)	5.95 (3.4-11.9)	0.699
Thrombocytes	242 (198–332)	311.5 (239-471)	**0.028**
Neutrophil granulocytes	3.30 (1.80–7.20)	3.2 (1.8-7.9)	0.747
Lymphocytes	2.05 (1.60–3.10)	2.35 (1.4-3.4)	0.364
Monocytes	0.50 (0.30–0.90)	0.5 (0.3-0.8)	0.735
Eosinophil granulocytes	0.24 (0.09–0.40)	0.13 (0-0.19)	**0.012**
Basophil granulocytes	0.04 (0.01–0.09)	0.05 (0-0.1)	0.601
High-sensitivity CRP	1.26 (0.16–9.28)	1 (0.21-9.54)	0.604
Ig-A	2.70 (0.69–3.30)	1.65 (0.63-3.7)	0.332
S-IgG	9.75 (8.20–13.40)	10.25 (7.5-16.5)	0.897
S-IgM	1.05 (0.35–1.30)	1.35 (0.59-2.9)	*0.079*
**Circulating cytokines**			
IFN-gamma	0.64 (0.64–5.13)	0.64 (0.64-16.38)	0.857
IL-1RA	4.06 (1.90–33.28)	3.56 (2.5-12.86)	1.000
IL-6	0.58 (0.32–1.87)	0.32 (0.32-2.25)	0.679
IL-8	1.25 (0.66–2.55)	1.08 (0.32-2.64)	0.401
IL-10	4.34 (3.02–6.95)	3.75 (1.28-11.52)	0.897
IL-17A	3.20 (3.20–19.18)	3.2 (3.2-13.06)	0.916
IL-18	47.23 (10.43–148.77)	67.67 (19.53-132.02)	0.245
TNF-α	2.77 (1.85–10.33)	3.2 (1.89-10.4)	0.121
TNF-β	0.80 (0.80–11.87)	0.8 (0.8-8.69)	0.916
TGF-β1	6345.36 (4155.44–40136.52)	8481.19 (4921.56-58895.29)	0.121
**Gene Expression (RGE)**			
IL-18	1.18 (0.75–2.23)	0.85 (0.36-1.47)	0.121
ILRN	1.03 (0.45–3.35)	0.83 (0.53-2.25)	0.897
TNF	1.14 (0.75–2.03)	0.98 (0.69-1.23)	0.302
IL-6	0.91 (0.52–1.67)	0.94 (0.53-2.11)	0.699
CXCL8	1.15 (0.61–2.13)	0.84 (0.39-2.21)	0.699
IL-10	1.03 (0.39–2.47)	1.24 (0.38-2.81)	0.156
IFN-γ	1.20 (0.58–3.02)	0.86 (0.19-2.28)	0.519
TGF-β1	1.07 (0.50–1.41)	1.01 (0.76-1.22)	0.606
IL-17	0 (0.00–0.01)	0 (0-0.01)	1.000
FOXP3	1.12 (0.82–1.31)	1.06 (0.54-1.26)	0.439
RORC	1.06 (0.45–1.69)	1.13 (0.45-1.69)	0.519
TBX21	1.38 (1.10–2.01)	0.84 (0.46-1.58)	**0.02**
GATA3	0.99 (0.82–1.88)	0.95 (0.45-1.3)	0.302

Note: Data are presented as median (minimum-maximum). p-values are assessed using Mann-Whitney *U* test comparing the schizophrenia and OCD group. Bold indicates statistical significance (p < 0.05). *Italics* indicates p-value <0.10.

Abbreviations: CRP. C-Reactive protein; Ig. Immunoglobulin, IFN. Interferon; IL. Interleukin; TNF. tumor necrosis factor; TGF. Transforming growth factor; RGE. relative gene expression.

**Fig. 2:**
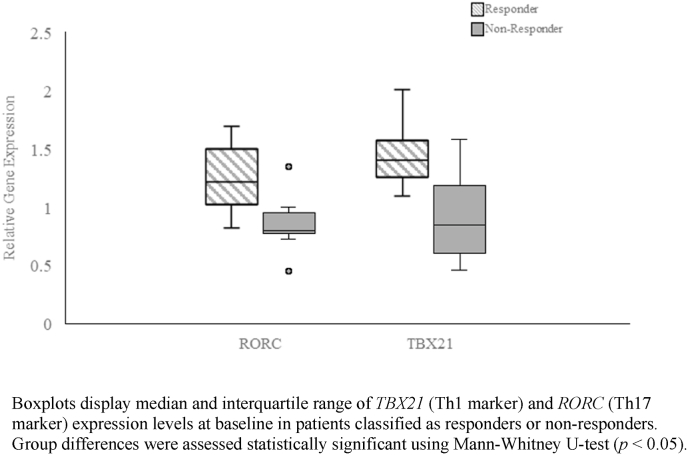
Baseline relative expression of TBX21 and RORC in responders and non-responders. Boxplots display median and interquartile range of *TBX21* (Th1 marker) and *RORC* (Th17 marker) expression levels at baseline in patients classified as responders or non-responders. Group differences were assessed statistically significant using Mann-Whitney *U* test (*p* < 0.05).

Focusing on cytokines and gene expression of inflammatory related proteins at baseline, the expression of *TBX21*, a transcription factor associated with the Th1 subset of CD4^+^ T cells, was significantly higher among responders than non-responders (Table 3). *TBX21* expression also showed a positive association with functional improvement, as higher baseline levels were associated with greater increases in PSP scores at week 12 (Spearman's ρ = 0.675, *p* < 0.008, Fig. 2). *RORC*, a transcription factor linked to Th17 cells, was also higher among responders (Table 3), but was not associated with PSP increase (ρ = 0.408, p = 0.148).

**Table 3:** Differences in responders vs non-responders.

	Responders (N = 6)	Non-response (N = 8)	p-value
**Clinical routine measurements**			
Leukocytes	6.45 (4.8-8.4)	6.9 (3.4-11.9)	0.699
Thrombocytes	242 (198-332)	322 (226-471)	*0.053*
Neutrophil granulocytes	3.3 (2.1-5.3)	4 (1-14)	0.796
Lymphocytes	2.05 (1.6-2.8)	2.3 (1.4-3.1)	0.194
Monocytes	0.45 (0.3-0.5)	0.5 (0.3-0.9)	0.176
Eosinophil granulocytes	0.24 (0.18-0.36)	0.13 (0-0.4)	**0.023**
Basophil granulocytes	0.05 (0.03-0.07)	0.05 (0-0.1)	0.601
High-sensitivity CRP	1.27 (0.16-9.28)	1.09 (0.16-9.54)	0.795
S-IgA	2.85 (1.4-3.3)	1.3 (0.63-3.7)	**0.033**
S-IgG	9.75 (8.6-11.5)	10 (7.5-13.6)	0.948
S-IgM	1.15 (0.35-1.3)	1.2 (0.59-2.5)	0.363
**Circulating cytokines**			
IFN-gamma	0.64 (0.64-5.13)	0.64 (0.64-6.28)	0.719
IL-1RA	4.06 (2.23-33.28)	3.48 (1.9-13.15)	0.897
IL-6	0.59 (0.32-1.87)	0.32 (0.32-2.25)	0.783
IL-8	1.25 (0.66-2.55)	1.39 (0.89-2.64)	0.561
IL-10	4.19 (3.02-6.95)	4.46 (1.28-11.52)	0.796
IL-17A	3.2 (3.2-3.2)	3.2 (3.2-19.18)	0.204
IL-18	47.23 (15.6-115.23)	72.11 (10.43-148.77)	0.245
TNF-α	2.78 (2.56-4.45)	3.75 (1.85-10.4)	0.156
TNF-β	0.8 (0.8-11.87)	0.8 (0.8-8.69)	0.751
TGF-β1	6345.36 (5563.08-9994.03)	13476.73 (4155.44-58895.29)	0.156
**Gene Expression (RGE)**			
IL-18	1.15 (0.75-1.47)	1.12 (0.54-2.23)	1.000
ILRN	1.03 (0.79-1.43)	0.92 (0.45-3.35)	0.897
TNF	1.14 (0.75-1.29)	0.99 (0.69-2.03)	0.606
IL-6	0.91 (0.63-1.67)	0.91 (0.52-2.11)	0.897
CXCL8	1.04 (0.61-1.57)	1.14 (0.51-2.21)	0.699
IL-10	0.68 (0.39-2.47)	1.57 (0.58-2.81)	0.245
IFN-γ	1.2 (0.58-2.25)	1.02 (0.24-3.02)	0.796
TGF-β1	1.07 (0.98-1.38)	1.08 (0.5-1.41)	0.606
IL-17	0 (0-0.01)	0 (0-0.01)	0.94
FOXP3	1.05 (0.82-1.3)	1.1 (0.67-1.31)	1.000
RORC	1.22 (0.82-1.69)	0.8 (0.45-1.35)	**0.028**
TBX21	1.4 (1.1-2.01)	0.85 (0.46-1.58)	**0.028**
GATA3	1.15 (0.86-1.88)	1.01 (0.45-1.3)	0.121

Note: Data are presented as median (minimum-maximum). p-values are assessed using Mann-Whitney *U* test comparing the response and non-responder group. Bold indicates statistical significance (p < 0.05). Italics indicates p-value <0.10.

Abbreviations: CRP. C-Reactive protein; Ig. Immunoglobulin, IFN. Interferon; IL. Interleukin; TNF. tumor necrosis factor; TGF. Transforming growth factor; RGE. relative gene expression.

In addition, Responders also showed higher baseline eosinophil counts and serum IgA levels compared with non-responders (Table 3).

**Fig. 3:**
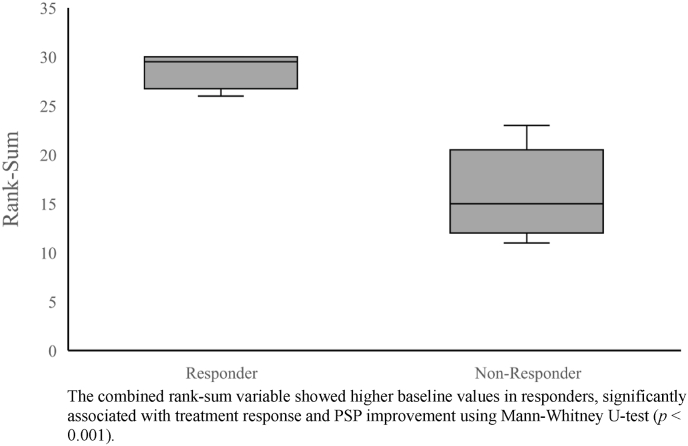
Baseline composite rank-sum of TBX21 and RORC expression in responders and non-responders. The combined rank-sum variable showed higher baseline values in responders, significantly associated with treatment response and PSP improvement using Mann-Whitney *U* test (*p* < 0.001).

Due the findings of differences in two different master regulators of T-cell differentiation (TBX21 and RORC), we found it important to consider possible joint effects of TBX21 and RORC in association to outcome. Since our sample was considered too small for formal interaction analyses, a composite variable was instead constructed *post hoc* for exploratory purposes from the sum of TBX21 and RORC relative gene expression, as respectively hierarchically ranked. This summarized variable was significantly higher in responders than non-responders (p < 0.001, Fig. 3) and also associated with PSP increase by week 12 (ρ = 0.793, p < 0.001).

Baseline levels of other biomarkers, such as interleukin-6 (IL-6), tumor necrosis factor-alpha (TNF-α) and high-sensitivity C-reactive protein (CRP), which have been focus of research in other studies (Orsolini et al., 2018), did not show any associations with treatment response.

### 3.3 Longitudinal changes in biomarkers at week 12

There were no significant differences between responders and non-responders in changes of cytokine or gene expression levels from baseline to week 12. However, TBX21 expression showed a trend toward a greater decrease among responders, with a median ΔTBX21 of −0.62 (range −1.06 – 0.00) compared with 0.04 (range −0.75 – 1.37) in non-responders (Mann–Whitney U, p = 0.089).

### 3.4 Sensitivity analyses

In a sensitivity analysis, we repeated the baseline biomarker comparisons within the schizophrenia subgroup only, comparing responders (n = 6) and non-responders (n = 2). Despite the very limited sample size, baseline RORC expression remained nominally higher in responders (median 1.21, range 0.82 – 1.69) than in non-responders (0.59, 0.45 – 0.72; p = 0.046). The post hoc composite rank-sum variable was also nominally higher in responders (median 29.5, range 26 – 30) than in non-responders (14.5, 11 – 18; p = 0.039). However, TBX21 did not show a statistically significant difference in the schizophrenia-only sensitivity analysis.

Since a difference between the schizophrenia and OCD group was identified for baseline level of TBX21, which emerged as interesting in the primary analysis, a post-hoc analysis was performed without exclusion of the two OCD participants responding up to 12 weeks. In this expanded sample, baseline RORC remained significantly higher in responders with a median of 1.23 (range 0.82 – 1.69) compared with 0.79 (0.45 – 1.35) in non-responders (p = 0.012). The composite rank-sum variable of TBX21 and RORC also showed a significant association with response, with higher values in responders than non-responders (median 27.50, range 21.00 – 30.00 vs. 14.00, range 5.00 – 23.00; p = 0.003).

None of the above associations on baseline or 12 weeks remained statistically significant after correction for multiple comparisons using the Benjamini-Hochberg method for false discovery rate (FDR).

To evaluate the potential for confounding in crude (i.e., unadjusted) analyses of *RORC* and *TBX21*, we examined univariate associations (correlations and group differences in dichotomized variables) of these two biomarkers with age, BMI, smoking status, and current antipsychotic treatment. None of these potential covariates were significantly associated with biomarkers, outcome variables (except for current antipsychotic treatment which was associated with schizophrenia diagnosis and outcome group) or each other when assessed individually, suggesting low risk of multicollinearity.

## 4 Discussion

In this exploratory study, we investigated associations between immunological biomarkers in plasma and treatment response to rituximab among patients with schizophrenia and obsessive–compulsive disorder (OCD). The overall results indicate that while cytokines did not differ between responders and non-responders, baseline expression of *TBX21* and *RORC,* two master regulators of T-cell response, was higher among schizophrenia participants with clinical improvement and functional gain. The consistency of these findings across outcome measures suggests a biological basis that warrants future exploration.

### 4.1 Role of TBX21 and RORC as biomarkers

*TBX21* (encoding the transcription factor T-bet) is a key regulator of Th1 differentiation and IFN-γ production, whereas *RORC* (encoding RORγt) drives Th17 cell lineage commitment. The compound variable constituting a rank sum of TBX21 and RORC showed the strongest association with response and the parallel involvement of these transcription factors may indicate that an imbalance in Th1–Th17 cell activity contributes to treatment response variability, and potentially to underlying disease mechanisms in schizophrenia.

The association between TBX21 and clinical response is particularly noteworthy given previous literature suggesting altered Th1 immune responses in schizophrenia. Patients with schizophrenia often display reduced production of type-1 cytokines, including IL-2 and IFN-γ, reflecting an imbalance or dysregulation of Th1 immunity (Müller and Schwarz, 2010; Kim et al., 2004). The observed association between higher *TBX21* expression and greater clinical improvement following rituximab may therefore indicate that individuals with relatively preserved Th1 activity constitute an immune profile more responsive to B-cell depletion. Rituximab, through depletion of CD20^+^ B cells, may indirectly attenuate aberrant Th1 activation and downstream neuroinflammatory signalling (Bar-Or et al., 2010; Hauser et al., 2008). However, it should be noted that TBX21 is also readily expressed in activated CD8^+^ T cells and in mature NK cells in whole blood, and future studies are needed to confirm whether increased TBX21 expression is associated solely with changes in Th1 cells or a more general increase in type 1 immunity (Huang and Bi, 2021).

The similar pattern observed for *RORC*, driving Th17 differentiation, suggests that Th17-related mechanisms may also contribute to treatment response.

In a recent review, Melnikov et al. summarise converging evidence that Th17 cells and their cytokines contribute to blood–brain barrier disruption, microglial activation and sustained neuroinflammatory signalling in schizophrenia, in parallel with dopaminergic and glutamatergic dysregulation (Melnikov et al., 2025; Nayak et al., 2025).

Melnikov et al. also highlight that circulating IL-17 concentrations are often low and variable, and that dysregulation of Th17 pathways may be more reliably captured at the level of cellular phenotypes or transcriptional programmes than by single cytokine measurements. This aligns with our observation that classical inflammatory markers such as IL-6, TNF-α and hsCRP did not differentiate responders from non-responders, whereas transcription factors associated with Th1 and Th17 differentiation (TBX21, RORC) showed robust baseline differences and predicted functional outcome. One plausible interpretation is that TBX21/RORC expression reflects a more stable, upstream dimension of T-helper cell polarisation, which in turn modulates how B-cell depletion reshapes pathogenic immune circuits.

Importantly, the interplay between *TBX21* and *RORC* is not mutually exclusive; *Th1-like Th17 cells* co-expressing T-bet (TBX21) and RORγt (RORC) have been described as a transitional or hybrid subset capable of producing both IFN-γ and IL-17.

Based on the observed associations for *TBX21* and *RORC* individually, we conducted a post hoc exploratory analysis to examine the association between a combined measure of these transcription factors and treatment response. The composite rank-sum variable was strongly associated with outcome compared with each biomarker analyzed separately, suggesting an additive or interactive contribution of Th1 and Th17 pathways.

Both Th1 and Th17 cells are known to promote neuroinflammatory cascades through secretion of pro-inflammatory cytokines and modulation of adaptive immune responses.

Th1 and Th17 cells also share effector mechanisms and display a high level of plasticity between each other. In multiple sclerosis, “hybrid” cells (Th17.1) have been shown to be more prone to infiltrate CNS than their mono-phenotypic counterpart (van Langelaar J et al., 2018), and Th1 and Th17 responses jointly contribute to disease through shared cytokines such as GM-CSF (Park, E., & Ciofani, M. 2025).

A combined and integrated measure may therefore better capture the complexity of immune dysregulation in the present schizophrenia sample. Supporting this notion, we also observed significant baseline differences in peripheral eosinophil counts and serum IgA levels between responders and non-responders, further indicating a broader immune involvement beyond changes in circulating cytokine levels.

Our findings, where both *TBX21* and *RORC* expression as well as their combined rank-sum were related to treatment response, support the notion that these transcriptional programs may jointly influence therapeutic outcomes. Even so, the direct translatability of these findings remains limited. Whole-blood qPCR is technically feasible in research, but several important steps would be required before TBX21 or RORC measurements could be considered for clinical use. Despite this clinical limitation, the present findings suggest that Th1- and Th17-related transcriptional signaling may represent biologically relevant pathways to investigate in future controlled studies of rituximab response in schizophrenia.

The absence of early clinical improvement in the OCD comparison group, despite similar exposure to rituximab, supports the notion that the specific immune-mediated mechanisms relevant to rituximab response in schizophrenia are not uniformly present across severe psychiatric disorders, but may represent a distinct inflammatory subtype. OCD patients may still respond (Gallwitz et al., 2025), but likely through partly different underlying mechanisms.

### 4.2 Analysis of biomarkers level change from baseline to week 12 after rituximab

Although the longitudinal analyses were limited by small sample size, the observed decreases in TBX21 and RORC expression among responders provide further support for the involvement of Th1- and Th17-related mechanisms in treatment response. These findings, while not statistically significant, align with the baseline associations and suggest that improvement may be accompanied by a downregulation of pro-inflammatory transcriptional activity.

This pattern could reflect rituximab's indirect immunomodulatory effects, whereby B-cell depletion dampens excessive T-cell activation and cytokine signalling. Such convergence between baseline predictors and within-subject changes strengthens the notion that *TBX21* and *RORC* are not merely correlates of disease severity but may participate in the immunological processes underlying therapeutic response. Future studies with repeated sampling in larger cohorts are warranted to further elucidate how modulation of Th1/Th17 activity contributes to symptom improvement in schizophrenia.

### 4.3 Limitations

Several limitations of this exploratory study should be acknowledged. The small sample size, particularly within the schizophrenia groupgeneralizability of the findings. The open-label design may introduce expectancy and observer bias, and the absence of a placebo or active control group prevents firm conclusions regarding rituximab's efficacy relative to standard treatment. However, in chronic or treatment-resistant samples of schizophrenia and severe OCD, treatment response rates are generally modest, and sustained clinical improvement is unlikely to be driven by placebo effects alone. (Siskind et al., 2022, Pallanti and Quercioli, 2006).

Another limitation concerns the composition of the comparison groups. The observed differences between responders and non-responders may partly reflect diagnostic heterogeneity rather than treatment response alone, as schizophrenia participants differed markedly from the OCD group in terms of baseline TBX21 levels, and SSD non-responders consisted of only two individuals. Although the Clinical Global Impression (CGI) scale and the Personal and Social Performance (PSP) scale are not specific for schizophrenia, the observed associations between PSP improvement and symptom reduction support the validity of our functional outcome measures, which are generic and relevant when comparing schizophrenia and OCD patients (Mavrogiorgou et al., 2016). This is in line with previous work demonstrating associations between peripheral inflammatory markers (e.g. C-reactive protein) and PSP scores in schizophrenia (Chumakov et al., 2022).

After correction for multiple testing, none of the results remained statistically significant, but with the small sample in mind we do not want to rule out biologically and clinically relevant associations and the findings are still deemed relevant from a hypothesis generating perspective.

Our small sample size clearly hampers the statistical power and use of statistical modelling suitable for larger samples, including interaction analyses, multiple regression or mixed effect models. However, we evaluated potential confounders—including age, BMI, smoking status, and ongoing antipsychotic medication—in univariate analyses and none showed significant associations with outcome variables, biomarkers or with each other, indicating no basis for major confounding effects.

### 4.4 Future directions

While the above limitations constrain interpretability, the consistency of observed immune-related patterns supports the biological plausibility of the findings. Larger, controlled studies are needed to validate these preliminary results and clarify the causal mechanisms linking immune modulation to symptom improvement. We are currently conducting a large double-blinded randomized controlled trial on rituximab for schizophrenia spectrum disorder (the RCT-Rits study with n = 120 participants, Bejerot S. et al., 2023b) where the hypotheses generated by the present study may be further evaluated. The current results suggest that mechanistic biomarkers as T-cell response key regulators such as TBX21 and RORC, may offer greater specificity for identifying patients likely to benefit from immunomodulatory treatment. This also underscores the need for integrative biomarker approaches in psychiatry.

## 5 Conclusion

In this explorative study we showed that an immunological profile characterized by elevated *TBX21* and *RORC* expression may constitute a marker for the mechanism behind responsivity to adjuvant treatment with rituximab in schizophrenia. A composite index combining these transcription factors increased the predictability, reflecting convergent Th1/Th17 pathway activity. If replicated and validated, this may strengthen previous findings of T-cell involvement in schizophrenia, and contribute to an elucidation of the mechanisms of immunomodulatory treatments in neuro-immune psychiatric syndromes.

Together, the baseline and longitudinal biomarker findings highlight the potential of integrating both clinical and molecular immune markers to improve prediction of treatment response. Further research should focus on validating our results and translating them to increase knowledge on underlying immune perturbations, thereby paving the way for future personalized psychiatric care.

Statement: During the preparation of this work the authors used ChatGPT 5.1 in order to check language cohesion of the text. After using this service, the authors reviewed and edited the content as needed and takes full responsibility for the content of the published article.

## Funding

This work was supported by ALF funding Region Örebro County; the Nyckelfonden [grant number OLL-878311 and OLL-779081]; the Torsten Söderbergs Foundation [grant number M84/19]; and the Swedish Brain Foundation [grant number FO2019-0094].

## CRediT authorship contribution statement

**Simon Bylund:** Conceptualization, Data curation, Formal analysis, Funding acquisition, Investigation, Methodology, Resources, Validation, Visualization, Writing – original draft, Writing – review & editing. **Daniel Eklund:** Conceptualization, Investigation, Methodology, Supervision, Writing – review & editing. **Mats B. Humble:** Conceptualization, Data curation, Methodology, Supervision, Writing – review & editing. **Susanne Bejerot:** Conceptualization, Data curation, Funding acquisition, Investigation, Methodology, Project administration, Resources, Supervision, Writing – review & editing. **Sverre Wikström:** Conceptualization, Investigation, Methodology, Supervision, Writing – review & editing.

## Declaration of competing interest

The authors declare that they have no known competing financial interests or personal relationships that could have appeared to influence the work reported in this paper.

## Data Availability

Data will be made available on request.
