# Supplementary material for: Th1/Th17-linked transcription factors TBX21 and RORC were associated with clinical response to rituximab in treatment-resistant schizophrenia
Source: Brain Behav Immun Health. 2026 May 27;54:101270. doi: 10.1016/j.bbih.2026.101270 (PMC13251702; doi:10.1016/j.bbih.2026.101270)
Supplement: Multimedia component 1 [file mmc1.docx]

**Supplemental tables**

Supplemental Table 2. Primers/probes with assay ID.

Gene Assay ID Type

IL18 Hs01038788_m1 Target gene

ILRN Hs00893626_m1 Target gene

TNF Hs00174128_m1 Target gene

IL6 Hs00174131_m1 Target gene

CXCL8 Hs00174103_m1 Target gene

IL10 Hs00961622_m1 Target gene

IFNG Hs00989291_m1 Target gene

TGFB1 Hs00998133_m1 Target gene

IL17A Hs00174383_m1 Target gene

FOXP3 Hs01085834_m1 Target gene

RORC Hs01076112_m1 Target gene

TBX21 Hs00894392_m1 Target gene

GATA3 Hs00231122_m1 Target gene

HPRT1 Hs02800695_m1 Reference gene

TBP Hs00427620_m1 Reference gene
